# Transactions of the Surgeon Apothecaries

**Published:** 1824-03-01

**Authors:** 


### *Transactions of the Surgeon Apothecaries. Vol.* 1.

[Continued from No. 15, page 550.]

Article III. *The History of a Case of Spontaneous Rupture of the Uterus in the Seventh Month of Pregnancy.* By Charles Shillito, M.R.C.S. and of the Society of Apothecaries.

Mr. Shillito is a gentleman of extensive practice, great zeal in his profession, and an accurate observer. We are glad, therefore, to find him an early contributor to these transactions; and we hope he will give them a steady support in future.

Rupture of the uterus, during labour, is one of the most unfortunate and melancholy occurrences that can be met with in practice. The fatality which almost invariably attends instances of this nature, evinces the insufficiency of our art; and whilst the feelings of the medical attendant are wounded in witnessing agony he cannot mitigate, his character too often suffers from the malignancy of prejudice, or the falsehood of ignorance. Happily, however, such accidents are rare; for nature has most admirably guarded against them, by causing the *tenacity* of the womb to increase with its distention, and its *strength* with the burden it has to bear.

The subject of this communication was housekeeper in an opulent family, in her 43d year; corpulent, but active:—had borne several children; and had a capacious and well formed pelvis.—One evening, in the 7th month of her 12th pregnancy, after more than usual exertion, symptoms of labour appeared;—some trifling gushes of blood escaped, and the motions of the child were strongly felt. The os uteri was found rigid, and admitting only the point of the finger.

No regular labour pains occurred until the following morning, when *two* very severe ones were experienced. The os uteri a little more dilated, but still hard. The patient complained of a heavy weight and coldness in the abdomen, and general uneasiness. The third day was ushered in by a rigor, followed by fever, intermitting pulse, increased restlessness, acute pain on deeply breathing, and tenderness and tumefaction of the abdomen, which increased with the day, attended by delirium, furred tongue, and anxiety of countenance. Dr. Merriman, who was now consulted, passed two fingers through the os uteri, and discovered a rent in the posterior part of the cervix. On the 4th day, Dr. M. got three fingers into the uterus—it was very much contracted, and contained only coagulated blood. A discharge, like lochia, took place on the 5th day, and spasms about the abdomen, bilious motions, copious vomiting of dark viscid secretion, rapid and intermitting pulse. These symptoms became more urgent, but were met by bold and judicious treatment; and the woman appeared to rally a little. A profuse discharge of dark, offensive, grumous matter continued to flow from the vagina until the 12th day, when a large quantity of water, with some pus, and the membranous part of the placenta with the funis, were discharged, after an attack of abdominal spasm. The child could now be plainly felt, through the muscles of the belly, resting on the brim of the pelvis from ilium to ilium. The patient was nearly in a state of syncope; her respiration was convulsive, and her pulse small and fluttering. "I got down" says Mr. Shillito "some volatile alkali diluted; also, by teaspoonfuls, strong brandy and water; and on her recovering a little, I took the opportunity of introducing my hand, with the navel-string for a guide. I found the os uteri apparently gone, and one common opening to the abdomen, and I felt a hand of the child low in the pelvis. After again supporting my patient by more stimuli, I a second time introduced my hand. I got hold of the feet, and, after half an hour's perseverance, being a little inconvenienced by the intestines pressing greatly upon my wrist, every movement of my hand causing eructations of wind, I extracted a perfect male fœtus in a discoloured and very putrid state, of the usual size of seven months growth. For several days after delivery, symptoms were as favorable as they could possibly be under such circumstances;—appetite, sleep, and strength appeared returning; but a change for the worse took place, and the poor woman sunk on the 25th day after the rupture of the uterus, and on the 12th after the delivery of the fœtus."

A minute description of the post mortem appearances is given by Mr. Shaw, with his usual accuracy. We must confine ourselves to that part of it most peculiarly connected with the case before us. The fœtus was found in a cavity beneath the peritonæum. "When the cavity was fully exposed, it appeared to have been of a size corresponding to the bulk of a seven month's fœtus. Its boundary on the upper part was formed by the adhesion of the left side of the arch of the colon to the abdominal muscles, extending in a line from the umbilicus to the 11th rib. The inferior boundary was formed by the natural reflection of the peritonæum from the fundus of the bladder, and which, on the left side, was continued up to the 11th rib, in connexion with the abdominal muscles; while on the right ride, the sac was closed by condensed peritoneum running from the umbilicus to the fundus of the bladder, so that the sac was confined principally to the left side of the abdomen. The posterior boundary was formed by the agglutination of the peritoneal surface of all the small intestines by masses of lymph."

"The vagina was not ruptured, but near the os tincæ it was in rather a sloughy state; only one half of the os tincæ was found, the other part having apparently sloughed off. The uterus was twice the size it is generally, in the unimpregnated state. The fundus and greater part of the body covered with the thickened and inflamed peritoneum; in other respects they appeared to be quite natural: but the left side of the os tincæ, of the cervix, and of part of the body of the uterus, appeared to have sloughed off, so that there was a direct communication between the cavity of the uterus and the sac in the abdomen. When the hand was passed into the vagina, if it were carried directly up, it entered into the sac in which the fœtus had lain, but by being directed laterally it passed into the uterus."

Mr. Shillito confesses he had no idea that the uterus was ruptured during the first forty hours of his patient's illness: but had such a circumstance been ascertained beyond a doubt, the rigid and contracted state of the os uteri precluded the possibility of relief by instrumental or manual interference.

The question, "Is it proper to deliver by artificial means in cases of ruptured uterus, or leave them to the efforts of nature?" has divided the opinions of writers on midwifery. Denman and Burns think every thing should be left to nature; whilst Dewees asserts such a procedure to be highly improper, and to diminish very much the chances of the patient's recovery. It appears to us, that both these opinions may be correct, each being the right one to follow under certain circumstances: and, that in adopting the one or the other, the medical man must, as in many other doubtful points of practice, be guided by his own judgement and experience, and the peculiarities of the individual case he has to do with.

Three plates are subjoined to this paper, illustrative of the morbid appearances discovered on dissection.

ARTICLE IV. *Cases in which certain kinds of Food commonly thought to be indigestible, have seemed to act as Violent Narcotic Poisons.* By CHARLES THOMAS HADEN, of Sloane Street, Surgeon to the Chelsea and Brompton Dispensaries.

Mr. HADEN states that the objects of this paper are;—"to show that food literally becomes poison under certain circumstances," and, that the action of food, and that of poison is the same, "differing only in degree."

That the effects of every thing taken into the stomach are modified or changed by the state of that organ and the general system, and that thus the same article of diet may at one time be grateful and salutary, and at another disgusting and injurious—that the same drug may be a bane or an antidote, are facts generally known and acknowledged; but, that the action of poison, which in its nature is destructive to the structure or functions of the body, and that of food, which is destined for its support and renovation, are the same, is a position the truth of which we think is very doubtful. We shall, however, leave this to the determination of our readers, referring them to the communication itself for a detail of the cases brought forward. They are examples where pulp of orange, fresh and pickled pork, beef's heart, mutton chops, &c. were apparently productive of the most alarming symptoms. We believe instances of the kind occur in the experience of most practitioners; and we are inclined, in some of the cases, to attribute a greater share of the effects produced to the *quantity* of what was taken, than our author appears to do.

Two cases of apoplexy are added:—in one, an old lady, repeatedly threatened with symptoms of the disease, experienced a fatal attack after mental agitation—the other, a young woman of 21, struck her head violently against a piece of wood, complained of head-ache for a day or two, and fell apoplectic on the fourth day after the accident, whilst standing at the wash tub. Appearances on dissection were, a slight congestion of scarlet blood, opposite the site of the blow;—all the ventricles filled with serum and blood—the cavity of the right nearly filled by a coagulum. A large clot had formed in the substance of the brain near the side of the third ventricle.

Article V. *Case of Injury to the Upper Jaw.* By John Powell, Esq. Keppel Street; Member of the Royal College of Surgeons.

A boy, eleven years of age, was thrown with great violence from a horse upon his head and face: he was stunned by the fall, and, on recovery, found the two front incisors of the upper jaw apparently knocked out, and a large wound in the under lip. In six weeks the wounded lip had healed, but several pieces of the upper jaw and palate came away at different periods, attended with fetid breath, and great obstruction to the passage of air through the nostrils. Ten years after the accident he became a patient of Mr. Powell's; his breath was, at that time, almost intolerable even to himself. Mr. P. found the obstruction in the nostrils to arise from the presence of the two incisor teeth, which had been driven by the force of the blow perpendicularly upwards into the right nostril. These were extracted with some difficulty, and the patient soon got well; and the two lateral incisors approximating, the general contour of the jaw was preserved without visible deformity.

Article VI. *On the Treatment of Painful Affections of the Nerves, arising from Local Injury.* By George R. Rodd, Esq. Surgeon, Hampstead, M.R.C.S.

Neuralgic affections are amongst the most distressing and painful complaints to which the complicated structure of man is liable, and often resist every remedial measure that theory, ingenuity, or experience can suggest. Mercury, steel, arsenic, lead, bark, opium, conium, stramonium, and belladonna, are but a few of the substances administered, here and there with success, but in very numerous instances, without any.

Mr. Rodd has been fortunate enough to relieve several cases by the external application of a strong watery solution of belladonna to the skin, over the affected nerve. In one instance the disease, which appeared to originate from a blow on the part, was in the sciatic nerve, extending from the hip to the foot—almost immediate relief was afforded by rubbing the course of the pain with two drams of the extract of belladonna dissolved in an ounce of water. The pain never recurred with much violence, and the woman got quite well by small doses, combined with extract of bark, and a blister at the back part of the thigh. Another case was that of a female, who suffered excruciating pain in the course of the sciatic nerve after parturition. It resisted every thing for six weeks, until the belladonna was applied.

Mr. Rodd thinks, that the weak and delicate who have suffered from hæmorrhage or other debilitating discharges, are most liable to painful affections of the nerves; and, that during a paroxysm, the function of the nerve is interrupted, which accounts for a thrilling sensation characteristic of this complaint. Pressure on a nerve will sometimes give rise to a derangement of its functions. Mr. R. suffered from this himself, from wearing a tight ring—of course, where its seat is beyond reach, no assistance can be derived from local applications.

Art. VII. *Case of Aneurism of the Aorta.* By John Hunter, Esq. Jun. Member of the College of Surgeons.

A sawyer, 48 years of age, complained of violent gnawing pains about the back, which were treated as rheumatic, but without even temporary benefit, excepting from bleeding and opium. They were then supposed to be hepatic, but the medicines administered had the same result. After a time the pains became more defined, fixing about the left shoulder. The hand applied to this and adjacent parts detected no pulsation or tumour;—there was no irregularity of pulse; no unusual beating in the chest. The poor man got no sleep; and was unable to take a deep inspiration without great increase of the pain. For a short time before his death his voice became hoarse, with a slight cough, with a numbness of the lower extremities, anorexia, and emaciation. He died of hæmoptysis.

*Dissection.* Eight or ten ounces of serum in the cavity of the pleura. "A quantity of florid blood extravasated in the lower edge of the right lung, and the pleura covering it highly vascular, (this was the immediate cause of death.") The lungs, with the exception of this, healthy. Heart pale and fat—left ventricle thin—three ounces of serum in the pericardium. "On removing the lungs an immense aneurismal tumour was discovered, proceeding from a circular orifice, above an inch in diameter, at the back of the aorta, about an inch and a half previous to the transmission of the arteria innominata; the posterior surface of the sac was formed by the six superior dorsal vertebræ, and portions of the corresponding ribs on either side; these bones were all in a state of ulceration." The sac had not burst;—the coagulum removed from it weighed two pounds ten ounces. The aorta, near the aneurism, had several large patches of ossification.

Article VIII. *Abstract of Communications from the Country on Medical and Surgical Attendance on the Parochial Poor.* By Robert Master Kerrison, M.D.

This, like most abstracts, admits not of analysis:—its nature, and our limits, oblige us, therefore, to refer to the communication itself.

ARTICLE IX. *A Series of Cases of bad Practice in Midwifery and Surgery, illustrative of the Evils which result from uneducated Persons being allowed to practise those Branches of the Medical Profession.* Collected by DAVID EVANS, Esq. Surgeon, of Belper, in Derbyshire.

It is certainly a national opprobrium, that ignorance should be suffered to sport with and destroy the lives of so many of our fellow creatures with impunity. Laws are passed to secure a man's property, and to bring to justice the thief who steals to the value of a shilling, whilst the knavish pretender to medicine is permitted not only to pick pockets to any extent, but to deprive a person of limb, of health, of life—without the fear of reprimand or punishment!

Almost every day's experience furnishes such instances as Mr. Evans relates: very many have fallen within the sphere of our observation, and doubtless within that of most of our readers. We sincerely hope the Society of Apothecaries will exercise that power which has been granted them by Parliament, with energy, promptness, and decision, and root out an evil which is spreading to a most alarming extent.

ARTICLE X. *A Case of Poisoning by Opium, in which the Stomach was successfully emptied by mechanical Means; also, a successful Case of Operation for an extraordinary Tumour found in the Vagina.* By DAVID EVANS, Esq. Surgeon, of Belper, Derbyshire.

A woman, 55 years of age, confined to bed from a paralytic state of the lower extremities, was in the habit of taking thirty or forty drops of tincture of opium in gin to alleviate pain. Being one day left with an ounce and a half of the former, and a pint of the latter, she swallowed the whole. She was found soon after with a ghastly and mottled countenance, livid lips, and dull and half-closed eyes—her breathing was slow, pulse quick, irregular, and feeble, and all the muscles relaxed. Deglutition apparently destroyed. Through a flexible tube passed down the œsophagus, half a dram of ipecacuanha in a little water, and soon after three pints of warm water, were injected. From the emaciation of the patient the distention of the stomach by this quantity of fluid was apparent; pressure was made over this organ, and its contents, smelling strongly of gin and opium, ran out through the tube. This operation was repeated until the peculiar odour disappeared. The patient now rallied a little, an emetic took effect, and she recovered from the influence of the opium; but died soon after of inflammation of the chest.

*A Case of Tumour in the Vagina, &c.*

A soldier's wife, in the third month of her pregnancy, slipt and fell to the ground. She felt at the time "as if she had displaced something within her." A train of symptoms, indicating abdominal inflammation, followed the accident; these were removed by bleeding, &c. but a state of feverish restlessness, and occasional uterine pains, led to an examination per vaginam. "I was astonished," says Mr. Evans, "to find a large tumour, the size of a child's head, hard and incompressible, and feeling like a bony and cartilaginous substance. It was connected with the upper and back part of the vagina," and so completely filled that passage that no part of the uterus could be felt. The tumour caused a suppression* of urine, was highly inflamed, and tender to the touch; and the belly was tumid and painful—a trochar was thrust into the tumour, and ℥ij. of a jelly-like fluid, tinged with blood, passed off. This gave considerable relief for 24 hours, when the painful symptoms returned in an alarming degree, with delirium, and appearances of approaching dissolution. An opening large enough to admit the finger, was made into the tumour with a scalpel; its walls were as hard as cartilage, and about half an inch in thickness. More of the jelly-like substance came slowly away. The finger passed far into the wound, detected several tumours, of various sizes, growing from the inner surface of the sac; some of which, somewhat like spermaceti, came away in a few days. The walls of the sac did not collapse at the time of the operation, but all the bad symptoms vanished, and the woman went her full time, when, after a "laborious labour," she was delivered of a dead child. At this time no vestige of the tumour remained.

Art. XI. *On Venous Congestion.* By Thomas Sandwith, Surgeon, Beverley.

Mr. Sandwith remarks that the diagnosis and treatment of the diseases of the mucous, serous, and cutaneous surfaces, constituting the greater part of the disorders of the human body, are, in general, well understood; whilst those situated in the substance of the brain, lungs, and liver, are often obscure. This arises from the peculiarity of the vascular structure of these organs: they are supplied with large venous re-

* Retention—*Rev.*

servoirs, in which, on a derangement in the just balance of the circulation, an undue accumulation of blood takes place, constituting congestion—a source of many important diseases.

1. *Congestion of the Brain.* The veins being passive organs, the motion of their blood depends upon the vis à tergo; congestion of the brain, therefore, is likely to happen when the arteries are atonic from debility or excitement. This assumes the characters of apoplexy, mania, delirium tremens, epilepsy, and hysteria, according to the predisposition of the individual.

Apoplexy, from congestion of the veins and sinuses, is not uncommon in young plethoric women who menstruate irregularly. *Acute* forms of *mania* depend on excitement of the arteries of the brain—the *chronic* species are often maintained by congestion of the veins—many unfortunate individuals oscillating between the two. The arteries of drunkards become atonic—hence venous congestion, producing delirium tremens. Syncope is the most complete form of venous congestion; and fainting from loss of blood is often attended with convulsions. It is not improbable, therefore, that epilepsy sometimes depends on congestion of the brain, as may hysteria, and many other diseases of the nervous system, as chorea, tetanus, and hydrophobia. Our author's experience does not enable him to decide this question; but, he thinks, the resemblance of hydrophobia to hysteria, as pointed out by Dr. Powel,* worthy of remark.

2. *Congestion in the Chest.* Mr. Sandwith considers angina pectoris as a striking example of venous congestion. A disease analogous to this affection occurs when the arterial system is weakened by profuse hæmorrhages—the right chamber of the heart and pulmonary veins suffering congestion in consequence. This often happens in those affections of the uterus, in which large sanguineous discharges alternate with leucorrhœa. Our author has been very successful in the treatment of these cases by repeated moderate bleedings, as suggested to him by Dr. Alderson, of Hull. There is a modification of this disease of the chest, in which the right auricle, pulmonary artery, and venous system of the liver, are overcharged. It is attended with feeble pulse; cold, pale, relaxed skin; a painful struggling about the heart, palpitations, shortness of breath, dyspepsia, and syncope. Cordials aggravate, but the lancet removes it. Hæmoptysis occurs from congestion of the pulmonary veins; most frequently in young females with suspended menses.

* Med. and Phys. Journal, vol. xx. p. 201.

3. *Congestion of the Liver.* This is the source of many serious diseases in remote parts. The disorders named bilious, dyspeptic, &c. arise from this. In its simplest form it is denoted by paleness of face, livor round the eyes, hepatic torpor, indigestion, and irregular bowels. An attack of melæna not unfrequently removes more advanced stages, or it is changed into inflammation. Under this cause may be arranged as effects hæmorrhoids, epistaxis, hæmatemesis, menorrhagia, purpura hæmorrhagica, bronchitis, phthisis, gout, and hypochondriasis.

The treatment of the different forms of congestion may be summed up in a few words. The object is to equalize the circulation. The oppressed veins must be relieved of part of their contents, and the warm bath, with frictions, counter-irritation, purgatives, occasional mercurials, and stomachic medicines are to be directed.

This paper indicates much talent for observation and reflection, and is certainly very creditable to Mr. Sandwith.

Article XII. *Narrative of an unusual Case of Utero-Gestation, in which the premature Expulsion of one Fœtus preceded, by two Months, the Birth of a Twin Fœtus at the full term.* By W. Newnham, Esq. Surgeon, Farnham, Surry.

In this instance a fœtus of seven months, with its placenta, were expelled from the uterus, still born, and another which remained behind, came away fifty-nine days after, being its full time, alive and well.

Mr. Newnham does not consider this as a case of superfœtation, but that two ova were impregnated at nearly the same time. It is an admirable provision of Nature, in cases of twins, that a dead fœtus should be an irritant to the uterus, cause it to take on the expulsive action for its exclusion, and then remain at rest to perfect the living one. The practical inference drawn from this narration is, that in premature labours where one fœtus is expelled we should not hasten the delivery of the other by any means, but tranquillize, and leave all to nature.

Article XIII. *Cases illustrative of the Efficacy of the Colchicum Autumnale.* By G. Wallis, M.D. and late of Emanuel College, Cambridge.

Every medicine must be tested by extensive experience and careful observation before its value can be justly estimated: we find, however, as Dr. Wallis observes, that when a remedy becomes *fashionable*, it is administered by the generality of practitioners "without reason and judgment," in all cases of disease, however opposite in their symptoms, and not being attended with universal success, it is condemned and discarded. This has been the case with many a valuable article of the materia medica, whose qualities deserved a better fate.

Dr. Wallis considers the powers of the colchicum, to be to diminish the excitability of the brain and nerves, and to lower the action of the heart and arteries. The powder is the form he has used it in. In a case of what is generally called rheumatic gout, seven grains combined with Dover's powder, administered every four hours, acted like a charm. Two cases of "hysteritis" from suppressed menses, which resisted the most active measures, readily yielded to this remedy. In pneumonia, pleuritis, phthisis, incipient hydrocephalus, and diabetes, it has been of infinite service. In this latter disease, its control over the urinary secretion was "extraordinary and intermediate" reducing it from three gallons, to its natural quantity, in two days.

Article XIV. *On the Treatment of a Peculiar Lameness produced by a Failure of the Arch of the Foot.* By Thomas Haden, Esq. Derby.

This lameness results from a defect in the arch, formed by the tarsal bones. From a weakness of the ligaments which bind these together, the arch gives way, the ankle sinks, the instep becomes flattened, the point of the heel raised, and the fore part of the os calcis, where it joins the astragalus, is depressed:—in some instances so much as to touch the ground. In consequence of this, the foot loses its elasticity, the toes are turned outward in progression, and the gait becomes laboured and awkward.

In treating this affection "nothing answered so well as broad strips of adhesive plaister rolled over each other, so as to form a compress of the proper width and thickness, and bound on the foot by means of other strips of plaister; the whole being fastened on and kept tight by a roller, or what is better, a laced stocking, or ankle-piece." "This will give no pain, if it be made flat enough at first: indeed, in the course of a day or two, it becomes a great comfort to the wearer. It should be changed once a week, and the compress then enlarged. This may be done by rolling the strip of plaister used to keep the compress in its place, on the old compress itself, and the increase of size thus given will generally be found the proper one for the time."

Article XV. *On the Treatment of Club Feet.* By Thomas Haden, Esq. Derby.

The treatment of this deformity has engaged the attention, and exercised the ingenuity of many. The knowledge of the surgeon has been aided by that of the mechanic, and yet how little success has attended their efforts! It is probable this may be accounted for by the various opinions which have existed respecting its cause. Mr. Haden attributes it in many instances to the pressure of the uterus in the last months of pregnancy. Ulcers of the foot, fractures of the leg, causing the patient to keep the part in an unnatural position, or injuries to the ankle joint, may produce this distortion.

The following is Mr. Haden's method of treatment. "For an infant, take a strip of plaister an inch broad, and nine inches long; place one end of it on the outside of the foot; carry the plaister over the middle of the instep, and down under the foot, so that it shall cover the end which lies on the outer edge of the boot, then twist the foot strongly, so as to turn the sole outwards instead of inwards, and secure it in that position, by carrying the plaister round the inner ankle to the outside of the foot. This plaister must be changed *every day*, and be farther kept in its place by means of a roller. Moreover, it is necessary at night to put both on the inside and outside of the leg and foot a strong splint of pasteboard, like a leg for drying stockings on, shaped at the bottom to the foot, and reaching up to the knee. These splints are to be removed every morning." Thus applied, sticking plaister will generally be sufficient for the removal of this deformity, as it answers every indication required, allows the limb to be moved in any direction, and is free from the weight and inconvenience of the more complicated apparatus. Some instances are mentioned of patients up to six years of age, who were cured in a few weeks.

Article XVI. *Brief Memoir on the Professional Character of William Richard Morel, late one of the Surgeons to the Westminster Hospital.* By his Colleague.

Who this Colleague is does not appear on the record. Be that as it may, we notice this brief memoir, out of its turn, in order to correct a *misrepresentation*, which it contains. If it be a proper maxim, to speak nothing but good of the dead, it is, we imagine, equally proper to speak nothing but truth of the living. Whether this last has been adhered to in the memoir, we leave our readers to judge when they have perused the following note, and the comment which we shall make upon it.

"He (Mr. Morel) was on the staff twenty years all but a few months: had he been allowed to remain there the proper time, he would have retired upon full-pay, which his active, arduous, and zealous, services justly entitled him to. But he was *obliged* to retire prematurely upon half-pay, having been informed that the hospital establishment was immediately to be broken up. Instead of which, the situation which he was thus induced to relinquish was given to another, and the hospital not broken up till after the time had expired which would have determined his rightful claims." 389.

The following are the *facts* of this hard and fictitious case.

Mr. Morel had served on full pay *only sixteen years.* Had he completed a service of twenty, he would not have been entitled to full pay; nor to any thing beyond the half pay at seven shillings a day, (which rate he received) unless the cause of reduction were ill health, when he might have claimed ten shillings a day.

The reduced establishment of medical officers required, that all those who were retained on full pay should be fit for any service; but Mr. Morel was much afflicted, and was in fact scarcely equal to his share of the duties that then devolved on the limited number of officers retained at the York Hospital.

In consideration of his broken down constitution, the heads of his department, made strenuous efforts with the Secretary at War, and Board of Treasury, to obtain for him a pension of 100*l.* a year: they had to regret want of success in their application; but Mr. Morel was very sensible of and expressed his gratitude for their exertions. On his retirement, he obtained the brevet rank of Deputy Inspector of Hospitals, as an honorable mark of approbation of his conduct as a medical officer.

So much for the *twenty years* service and *forced* retirement of Mr. Morel—and harsh conduct of the heads of the Army Medical department!

There are several important memoirs remaining to be analyzed in this volume, but these we must defer till our next number.

